# A mathematical analysis of an extended MHD Darcy–Forchheimer type fluid

**DOI:** 10.1038/s41598-022-08623-0

**Published:** 2022-03-28

**Authors:** José Luis Díaz Palencia

**Affiliations:** Escuela Politécnica Superior, Universidad Francisco de Vitoria, Ctra. Pozuelo-Majadahonda Km 1,800, Pozuelo de Alarcón, 28223 Madrid, Spain

**Keywords:** Mathematics and computing, Physics

## Abstract

The presented analysis has the aim of introducing general properties of solutions to an Extended Darcy–Forchheimer flow. The Extended Darcy–Forchheimer set of equations are introduced based on mathematical principles. Firstly, the diffusion is formulated with a non-homogeneous operator, and is supported by the addition of a non-linear advection together with a non-uniform reaction term. The involved analysis is given in generalized Hilbert–Sobolev spaces to account for regularity, existence and uniqueness of solutions supported by the semi-group theory. Afterwards, oscillating patterns of Travelling wave solutions are analyzed inspired by a set of Lemmas focused on solutions instability. Based on this, the Geometric Perturbation Theory provides linearized flows for which the eigenvalues are provided in an homotopy representation, and hence, any exponential bundles of solutions by direct linear combination. In addition, a numerical exploration is developed to find exact Travelling waves profiles and to study zones where solutions are positive. It is shown that, in general, solutions are oscillating in the proximity of the null critical state. In addition, an inner region (inner as a contrast to an outer region where solutions oscillate) of positive solutions is shown to hold locally in time.

## Introduction

Non-Newtonian fluids are typical in different areas of sciences, from engineering applications to biomedical scenarios. The constitutive relations (particularly the description of an appropriate viscosity) require for a non-Newtonian fluid may lead to specific formulations supported (but probably not limited) to the classical descriptions of Navier-Stokes equations. As a consequence, the resulting differential equations typically involve non-linear terms that require specific analytical and experimental approaches. As a set of representative examples on the exposed generalities, the reader can refer to references^1–5^.

The Darcy–Forchheimer flow emerges as a kind of non-Newtonian fluid with specific rheological properties in applications involving porous media. Darcy–Forchheimer flow is been a source of research currently; as an example, in^6^, the heat transfer properties of a Darcy–Forchheimer flow in nanofluids with radiation are explored. In addition, in^7^ the authors study the convective flow of an MHD nanoliquid in an odd-shaped cavity filled with carbon nanotube-iron. In both cases, the modelling equations were provided in accordance with the well known Forchheimer model that is described some lines later.

Furthermore, the Darcy–Forcheimer fluid has been widely employed in soil pollution, nuclear reactors cooling, tumor growth dynamics, porous catalysis... For a complete set of discussions and applications of Darcy–Forchheimer flows, the reader is referred to^8–16^.

Generally, a 1-D Darcy-Forchheimer flow is governed by the following set of equations:

1 $$\begin{aligned} {\mathbf {V}}= & {} \left( v\left( y,t\right) ,0\right) , \, \, \, \, \, \mathrm{div} {\mathbf {V}}=0 \rightarrow \frac{\partial v}{\partial x} = 0. \end{aligned}$$

2 $$\begin{aligned} \frac{\partial v}{\partial t}= & {} -\frac{1}{\rho }\frac{\partial P}{\partial x}+\nu \frac{\partial ^{2}v}{\partial y^{2}}-\left( \frac{\sigma B_{0}^{2}}{\rho }+ \frac{\phi \nu }{K}\right) v-\frac{F}{\rho }v^{2},\nonumber \\ \ \ v(y,t)= & {} v_{0}(y). \end{aligned}$$

*P*(*x*, *y*, *t*) is the pressure distribution on a plane, $\nu =\frac{\mu }{\rho }$ refers to the kinematic viscosity, *F* the non-uniform inertia coefficient in the porous medium, $\Phi$ is the porosity, $\rho$ the fluid density, $\mu$ the dynamic viscosity and *K* the medium permeability.

Firstly, consider the derivation with regards to *x* in expression (2), so that:

$$\begin{aligned} -\frac{1}{\rho }\frac{\partial ^{2}P}{\partial x^{2}}=0. \end{aligned}$$

Upon integration, it holds: $-\frac{1}{\rho }\frac{\partial P}{\partial x}=K_{1}$. Then the Darcy-Forchheimer model in Eq. (2) reads:

3 $$\begin{aligned} \frac{\partial v}{\partial t}=K_{1}+\nu \frac{\partial ^{2}v}{\partial y^{2}} -\left( \frac{\sigma B_{0}^{2}}{\rho }+\frac{\phi \nu }{K}\right) v-\frac{F}{ \rho }v^{2}. \end{aligned}$$

Based on the Darcy–Forchheimer model driven by Eq. (3), it is the intention to provide an extended model introduced under certain theoretical approaches. Previously, it is the intention to provide a historical context in what regards with the different analytical paths followed along this article.

To start with, consider the seminal work firstly introduced by Kolmogorov, Petrovskii and Piskunov^17^, in combustion theory, and by Fisher^18^, in biology to predict genes interaction. These works introduced key ideas on how to face non-linear reaction–diffusion equations. As a basic principle, solutions were formulated under the scope of an incipient theory known as Travelling waves dynamics. The main question was focused on the conversion of a Partial Differential problem into an Ordinary Differential one, but it introduced a philosophical concern on the way solutions behave. Indeed, it was observed that depending on the selected Travelling wave speed, solutions may be oscillating or monotonic. Then, the question was mainly focus on selecting an appropriate travelling speed replicating the correct profile behaviour. Since the first formulation, the Fisher-KPP model has been applied to different ubiquitous applications: from ecology or biology (refer to^19–21^) to non-linear fluids^22^.

The proposed problem along this article can be named as an Extended Darcy–Forchheimer, in the sense that further theoretical terms are introduced on the classical formulation in Eq. (3). Firstly, an heterogeneous diffusion is introduced to account for potential heterogeneities in the proximity of the null critical state. Such heterogeneous diffusion is formulated with a high order operator. As an example on a process deriving into a fourth order operator, the reader can refer to^23^) where the authors proposed an energy approach to understand the patterns formation in materials. In addition, high order operators have been of wide use in physics to control unstable patterns in the proximity of critical points given by bistable systems (refer to^24–26^). Mathematically, such operators are an intense area of research. As a representative example, the Giorgi’s conjecture has been proved for an Allen-Cahn equation formulated with a high order operator (see^27^). The fact of introducing heterogeneous diffusion shall be contemplated in accordance with the problem particularities to predict. Along this analysis, a fourth order operator has been admitted, nonetheless other possible diffusion types can be assumed depending of the reality to model (for instance, refer to the p-Laplacian approach in^28^ together with the p-Laplacian non-Lipschitz^29^). Finally and in relation with the advection term introduced, some interesting results to show well-posedness of solutions have been proved by Montaru in^30^.

Based on the last discussion, the proposed problem (*P*) is formed of three main terms: a heterogeneous diffusion in the form of a high order operator, a non-linear advection and a non-uniform reaction term of Darcy–Forchheimer type.

4 $$\begin{aligned} \begin{array}{c} \frac{\partial v}{\partial t} = - \frac{\partial ^4 v}{\partial y^4} + c \, \frac{\partial v^q}{\partial y} + |y|^\mu v(-A-B \, v),\\ v_0(x) \in L_{loc}^2(\mathbb {R}) \cap L^\infty (\mathbb {R}), \\ v_{0,x} \in L^q(\mathbb {R}), \\ c \in \mathbb {R}, \, \, \, \, \, q> 1, \, \, \, \, \mu > 0, \, \, \, \, A= \left( \frac{\sigma B_{0}^{2}}{\rho }+\frac{\phi \nu }{K}\right) , \, \, \, \, \, B = \frac{F}{\rho }. \end{array} \end{aligned}$$

As discussed, the introduction of a high order operator aims the modelling of heterogeneous diffusion in a given media. Each type of fundamental non-linear diffusion has certain properties of potential interest in modelling. For instance, the Porous Medium Equation and the p-Laplacian driven diffusion have the property known as finite propagation along compact supports in finite mass functions. In this case, the high order diffusion induces a set of oscillating solutions whose properties permit to model diffusion in non-homogeneous media. Furthermore, the introduction of a non-linear advection has the objective of modelling specific flow movement induced by external means and encountered through the coefficients *c*, *q*. The non-uniform reaction depends on the spatial variable *y* through the term $|y|^\mu$ and is postulated to model the environmental medium variation. Indeed, along the integration domain *y*, the media can change leading to a temporal change in the velocity (fluid local acceleration). Note that many functions can be provided instead of $|y|^\mu$, nonetheless the proposed one permits to account for radial topological variations typical of annular geometries.

The search of precise profiles of solutions is given in the travelling wave domain. This will permit to account for a system of ordinary differential equations, with certain further considerations of an appropriate travelling wave speed in combination with the other parameters introduced $q, c, \mu$. Note that the travelling waves approach to non-linear reaction and diffusion models have been considered in different scenarios. The reader can refer to^31–35^ . The mathematical analysis along this paper starts with the formulation of the problem as an abstract evolution supported by a strongly continuous semigroup. This permits to account for the analysis of regularity, existence and uniqueness of solutions. Afterwards, instability of solution profiles are studied in the Travelling wave domain. To this end, a set of Lemmas are introduced supported by the definition of generalized norms in a Hilbert–Sobolev space. Afterwards, a numerical exploration provides different types of solution profiles for different values in the involved model parameters $c, q, \mu$. To make the numerical exploration possible, the solver bvp4c in MATLAB is used. Such solver is based on an implicit Runge-Kutta algorithm with interpolant extensions^36^ together with a collocation method at the borders.

## Bound properties, existence and uniqueness

### Previous definitions

#### Definition 1

Consider the following weighted Sobolev space $\, H^4_\alpha (\mathbb {R})$:

5 $$\begin{aligned} \Vert v\Vert _\alpha = \int _{\mathbb {R}} \alpha (\delta ) \sum _{k=0}^4 |D^v v(\delta )|^2 d \delta , \end{aligned}$$

such that $D=\frac{d}{d \delta }$, and $v \in ( H^4_\alpha \cap W^{4,2})(\mathbb {R}) \subset L^2_{\alpha } (\mathbb {R}) \subset L^2 (\mathbb {R})$ by Sobolev embedding. Note that the weighing function $\alpha (\delta )$ is defined according to ideas exposed in^30,35^:

6 $$\begin{aligned} \alpha (\delta ) = e^{a_0 \vert \delta \vert ^{\frac{4}{3}} - a_1 |\delta |^\mu - \frac{1}{\delta ^q} \frac{1}{\tau ^{\Phi }} \int _0^\tau (\Vert v_y(\tau )\Vert ^q + 1) d\tau }. \end{aligned}$$

Where the constant $a_0 > 0$ is sufficiently small, $0<a_1<<1$ is determined in a local time for $|v(y,t_0)|<<1$ (so as to make dominant the exponential term $e^{-a_1 |\delta |^\mu }$) and $\Phi > q+1$.

In addition, a mollifying norm is introduced. This is a relevant definition as the exponential mollifier permits to study possible patterns of extreme oscillating solutions.

#### Definition 2

Given $j \in \mathbb {R}^+$ and given $0 \le t < \infty$, the mollifying space $H^j$ is defined as per the norm:

7 $$\begin{aligned} \Vert v \Vert _{H^j} = \int _{-\infty }^{\infty } e^{j \delta ^2} \, |\hat{v}(\delta , t)|^2 d\delta , \end{aligned}$$

where the exponential kernel satisfies the $A_p$-condition for $(p=1)$^37^.

### Regularity and bound of solutions

Firstly, consider the fundamental problem:

8 $$\begin{aligned} v_t = Lv, \end{aligned}$$

where *L* is a general operator in $\mathbb {R}^N$ (including with no loss of results, $N=1$):

9 $$\begin{aligned} L =( - \Delta ^2 + q v^{q-1} c \cdot \nabla ). \end{aligned}$$

Then, the following bound properties hold:

#### Lemma 2.1

*For* $v_0 \in L^2(\mathbb {R})$, *the following bound holds*:

10 $$\begin{aligned} \Vert v\Vert _{L^2} \le \Vert v_0\Vert _{L^2}. \end{aligned}$$

*Admit* $$v_0 \in L^2(\mathbb {R}) \cap H^j(\mathbb {R})$$, *then*:

11 $$\begin{aligned} \Vert v\Vert _{H^j} \le \Vert v_0\Vert _{H^j}, \, \, \, \, \, \, \Vert v\Vert _{H^j} \le \Vert v_0\Vert _{L^2}^2, \, \,\, \, \,for \, \, \, \, t \ge \frac{j}{4}. \end{aligned}$$

*In addition, the following holds*:

12 $$\begin{aligned} \Vert v\Vert _{\alpha } \le \Upsilon \Vert v\Vert _{H^j} \le \Upsilon \Vert v_0\Vert _{H^j}, \, \, \, \, \, \Upsilon = 25 \, \max \lbrace v, D^1v, D^2v, D^3v, D^4v \rbrace \end{aligned}$$

#### **Proof**

Given the fundamental equation (8), an abstract evolution is represented as:

13 $$\begin{aligned} v(y,t) = e^{tL} v_0(y). \end{aligned}$$

Performing the Fourier transform ($f-$ domain):

14 $$\begin{aligned} \hat{v}(f,t) = e^{t ( - f^4 + q \hat{v}^{q-1} c f i)} \, \hat{v}_0 (f). \end{aligned}$$

Then:

15 $$\begin{aligned} \begin{aligned} \Vert v\Vert _{L^2}^2&= \int _{-\infty }^{\infty } |e^{t ( - f^4 + q \hat{v}^{q-1} c f i)}|^2 \, |\hat{v}_0 (f) |^2 df= \int _{-\infty }^{\infty } e^{- 2 f^4 t} |\hat{v}_0 (f) |^2 df \\&\le \sup _{f \in \mathbb {R}} ( e^{-2 f^4 t}) \int _{-\infty }^{\infty } |\hat{v}_0 (f) |^2 df= \Vert v_0\Vert _{L^2}^2. \end{aligned} \end{aligned}$$

Then, $$\Vert v\Vert _{L^2} \le \Vert v_0\Vert _{L^2}$$.

Now, admit the mollifying—weighted Sobolev space $H^j$ with the norm (Eq. 7):

16 $$\begin{aligned} \begin{aligned} \Vert v\Vert _{H^j}&= \int _{-\infty }^{\infty } e^{j f^2} |\hat{v}(f, t)|^2 df = \int _{-\infty }^{\infty } e^{j f^2} |e^{t ( - f^4 + q \hat{v}^{q-1} c f i)}|^2 \, |\hat{v}_0 (f) |^2 df \\&\le \sup _{f \in \mathbb {R}} ( e^{- 2 f^4 t}) \int _{-\infty }^{\infty } e^{j f^2}|\hat{v}_0 (f) |^2 df= \Vert v_0\Vert _{H^j}. \end{aligned} \end{aligned}$$

Note that as per initial assumption $v_0 \in L^2(\mathbb {R})$, then:

17 $$\begin{aligned} \Vert v\Vert _{H^j} = \int _{-\infty }^{\infty } e^{j f^2} |\hat{v}(f, t)|^2 df \le \sup _{f \in \mathbb {R}} ( e^{j f^2} \, e^{- 2 f^4 t}) \int _{-\infty }^{\infty } |\hat{v}_0 (f) |^2 df. \end{aligned}$$

After standard arrangements:

18 $$\begin{aligned} \Vert v\Vert _{H^j} \le \left( \frac{j}{4t} \right) ^{1/2} \Vert v_0\Vert _{L^2}^2. \end{aligned}$$

To conclude:

19 $$\begin{aligned} \Vert v\Vert _{H^j} \le \Vert v_0\Vert _{L^2}^2, \end{aligned}$$

for any given $t \ge \frac{j}{4}$.

Similarly, admit the weighted Sobolev space $H_\alpha ^4$:

20 $$\begin{aligned} \Vert v\Vert _\alpha = \int _{\mathbb {R}} \alpha (\delta ) \sum _{k=0}^4 |D^k v(\delta )|^2 d \delta \le \int _{\mathbb {R}} e^{j \delta ^2} \sum _{k=0}^4 |D^k v(\delta )|^2 d \delta \le \Upsilon \int _{\mathbb {R}} e^{j \delta ^2} |v(\delta )|^2 d \delta \le \Upsilon \Vert v\Vert _{H^j}, \end{aligned}$$

being $\Upsilon = 25 \, \max \lbrace v, D^1v, D^2v, D^3v, D^4v \rbrace$. Note that this last variable definition relies on the Holder continuous inclusion of any Sobolev space (refer to^38, p. 79^). Then, it is concluded on the regularity of the derivatives up to the third order while the fourth order derivative shall be considered as a control parameter. The mollifying norm can be used as a bounding condition provided the fourth derivative is finite, leading to a finite $\Upsilon$.

We have proved that the space of mollifiers $H^j$ is bounded by the Lebesgue $L^2$. In addition, the Sobolev space of oscillating solutions $H_\alpha ^4$ has been shown bounded by the mollifying space $H^j$ upon a scaling $\Upsilon$. $\square$

The high order linear spatial operator $-\Delta ^2$ (or in $\mathbb {R}$, $D_x^4$) can be regarded as the infinitesimal generator of a one parametric (being the parameter $\tau$ with $0<\tau \le t$) strongly continuous semi-group. Based on this, the following abstract form holds (note that the formulation is provided in $\mathbb {R}^N$ for $N = 1, 2, 3...$):

21 $$\begin{aligned} v(\tau ) = e^{-\Delta ^2 \tau } v_0 + \int _0^\tau \left[ c \cdot \nabla e^{-\Delta ^2 (\tau -s)} v^q(s) + e^{-\Delta ^2 (\tau -s)}|y|^\mu v(s)(-A-B \, v(s)) \right] ds. \end{aligned}$$

A solution to the fundamental $v_t = -\Delta ^2 v$ with Dirac pulse as initial data $v(y,0) = \delta (y)$ is given by:

22 $$\begin{aligned} \hat{v}(t) = e^{-f^4 t} \hat{v}_0. \end{aligned}$$

A kernel *N*(*y*, *t*) is determined making the inverse transformation:

23 $$\begin{aligned} N(y,t)= F^{-1} (e^{-f^4 t}) = \frac{1}{2 \pi } \int _\mathbb {R} e^{-f^4 t - if y} df = \int _\mathbb {R} e^{-f^4 t}cos(f y) df. \end{aligned}$$

The last integral is convergent for $f \in \mathbb {R}$. As a consequence, a kernel *N*(*y*, *t*) exists. Based on such kernel the following mapping is defined in the space $H_\alpha ^4$:

24 $$\begin{aligned} \Sigma _{v_0, \tau }: H_\alpha ^4(\mathbb {R}) \rightarrow H_\alpha ^4 (\mathbb {R}), \end{aligned}$$

such that for $0 < \tau \le t$, it holds:

25 $$\begin{aligned} \Sigma _{v_0, \tau }(v) = N(y,\tau ) * v_0(y) + \int _0^\tau \left[ c \cdot \nabla N(y,\tau - s) * v^q(y,s) + N(y,\tau - s)*\lbrace |y|^\mu v(y,s)(-A-B \, v(y,s)) \rbrace \right] ds. \end{aligned}$$

The coming theorem provides regularity conditions for the mapping $\Sigma _{v_0, \tau }(v)$.

#### Theorem 2.1

*The single parametric operator* $\Sigma _{v_0, \tau }$ (*being* $\tau$ *the free parameter*) *is bounded in the space* $H_\alpha ^4(\mathbb {R})$ *with the norm* (*Eq*. 5).

#### **Proof**

As a previous first step, the following inequality $B_0 \Vert v_0\Vert _\alpha \le \Vert v\Vert _\alpha$ (being $B_0 > 0$ a suitable constant) is shown:

26 $$\begin{aligned} \begin{aligned} \Vert v\Vert _\alpha&= \int _{\mathbb {R}} \alpha (\delta ) \sum _{k=0}^4 |D^k \hat{v}(\delta )|^2 d\delta = \int _{\mathbb {R}} \alpha (\delta ) \sum _{k=0}^4 |D^k \left[ e^{t ( - \delta ^4 + q \hat{v}^{q-1} c \delta i)} \, \hat{v}_0 \right] |^2 d\delta \\&\ge \int _{\mathbb {R}} \alpha (\delta ) \sum _{k=0}^4 |D^k \left[ e^{t ( - \delta ^4 + q \hat{v}^{q-1} c \delta i)} \right] |^2 \sum _{k=0}^4 |D^k \hat{v}_0|^2 d\delta \ge B_0 \int _{\mathbb {R}} \alpha (\delta ) \sum _{k=0}^4 |D^k \hat{v}_0|^2 d\delta = B_0 \Vert v_0\Vert _\alpha . \end{aligned} \end{aligned}$$

Consider $B_0 = \inf _{\delta \in B_r} \lbrace \sum _{k=0}^4 |D^k \left[ e^{t ( - \delta ^4 + q \hat{v}^{q-1} c \delta i)} \right] |^2 \rbrace > 0$ and arbitrary small in $B_r = \lbrace \delta , \, \, |\delta | < r \rbrace$ for $r > 0$.

Now, returning to the single mapping $\Sigma _{v_0, \tau }$:

27 $$\begin{aligned} \begin{aligned}{}&\Vert \Sigma _{v_0, \tau } (v)\Vert _\alpha = \Vert \Sigma _{v_0, \tau }\Vert _\alpha \,\Vert v\Vert _\alpha \le \Vert N\Vert _\alpha \, \Vert v_0\Vert _\alpha + \int _0^\tau \left[ \Vert c \cdot \nabla N\Vert _\alpha \, \Vert w^q\Vert _\alpha + \Vert N\Vert _\alpha \, \Vert |y|^\mu \Vert _\alpha \Vert v\Vert _\alpha \, \Vert A+Bv\Vert _\alpha \right] ds\\&\quad \le \left[ \Vert N\Vert _\alpha \, \frac{1}{B_0\, t} + \int _0^\tau \left[ \Vert c \cdot \nabla N\Vert _\alpha \, \Vert v_0^{q-1}\Vert _{H^j} + \Vert N\Vert _\alpha \Vert |y|^\mu \Vert _\alpha \, \ \Vert A+Bv\Vert _\alpha \right] ds \right] \, \, t \,\Vert v\Vert _\alpha \end{aligned} \end{aligned}$$

where we have made use of inequality (see Eqs. (16) and (20)):

28 $$\begin{aligned} \Vert v^{q-1}\Vert _{\alpha } \le \Vert v^{q-1}\Vert _{H^j} \le \Vert v_0^{q-1}\Vert _{H^j} \end{aligned}$$

Based on the inequality (Eq. 27) and the convergence of the kernel *N*(*y*, *t*) shown in Eq. (23), we conclude that the right expression (27) is bounded. Hence, the single parametric operator $\Sigma _{v_0, \tau }$, $\tau >0$ is bounded as intended to show. $\square$

### Uniqueness

To show uniqueness, the following theorem holds:

#### Theorem 2.2

*The single parametric mapping* $\Sigma _{v_0, \tau }$ *as defined in Eq*. (24) *has a unique fix point, i.e.* $v(y,t) = \Sigma _{v_0, \tau }(v(y,t))$.

#### **Proof**

To show the proposed Theorem, consider:

29 $$\begin{aligned} \begin{aligned}{}&\Vert \Sigma _{v_0, \tau }(v_1)-\Sigma _{v_0, \tau }(v_2)\Vert _\alpha \le \int _0^\tau \Vert c \cdot \nabla N(x,\tau - s) * (v_1^q-v_2^q)+ \\&N(x,\tau - s)*[v_1(A+Bv_1)-v_2(A+Bv_2)]\Vert |y|^\mu \Vert _\alpha \Vert _\alpha ds \\&= \int _0^\tau \Vert \int _\tau ^s \lbrace c \cdot \nabla N(x,\tau - s-\nu ) (v_1^q-v_2^q)+N(x, \tau - s-\nu )[v_1(A+Bv_1)-v_2(A+Bv_2)] \Vert |y|^\mu \Vert _\alpha \rbrace d\nu \,\Vert _\alpha ds \\&\le \int _0^\tau \int _\tau ^s \lbrace \Vert c \cdot \nabla N(x,\tau - s-\nu ) (v_1^q-v_2^q)\Vert _\alpha + \Vert N(x, \tau - s-\nu )[v_1(A+Bv_1)-v_2(A+Bv_2)]\Vert _\alpha \Vert |y|^\mu \Vert _\alpha \rbrace d\nu \,ds \\&= \int _0^\tau \int _\tau ^s \lbrace \Vert c \cdot \nabla N(x,\tau - s-\nu )\Vert _\alpha \Vert v_1^q-v_2^q\Vert _\alpha + \Vert N(x, \tau - s-\nu )\Vert _\alpha \Vert v_1(A+Bv_1)-v_2(A+Bv_2)\Vert _\alpha \Vert |y|^\mu \Vert _\alpha \rbrace d\nu \,ds \\&\le \Lambda \int _0^\tau \int _\tau ^s \lbrace \Vert v_1^q-v_2^q\Vert _\alpha + \Vert v_1(A+Bv_1)-v_2(A+Bv_2)\Vert _\alpha \Vert |y|^\mu \Vert _\alpha \rbrace d\nu \,ds, \end{aligned} \end{aligned}$$

*N* and $\nabla N$ are bounded. Indeed, both are convergent functions in $\mathbb {R}$ (see Eq. (23)), then:

30 $$\begin{aligned} \Lambda = \sup \lbrace \Vert N(x, \tau - s-\nu )\Vert _\alpha \, , \Vert c \cdot \nabla N(x,\tau - s-\nu )\Vert _\alpha ; \, \, x \in \mathbb {R}, \, \, \, \forall t>0 \,, \, \, \rbrace \end{aligned}$$

for any value in the involved parameters $s,\nu$.

Each of the involved integrals in Eq. (29) shall be assessed. Previously, the following supporting function is defined:

31 $$\begin{aligned} A (\epsilon , s) = \left\{ \begin{array}{c} \frac{v_1(\epsilon , s)^q - v_2(\epsilon , s)^q}{v_1(\epsilon ,s)-v_2(\epsilon ,s)}\, \, for \, \, \, v_1 \not \equiv v_2\\ q v_1^{q-1} \, \, otherwise \\ \end{array} \right\} , \, \, \, \, \end{aligned}$$

For two fixed values of $\epsilon$ and $s=T$, the previous supporting function is bounded:

32 $$\begin{aligned} 0 \le A(\epsilon ,s) \le c_0(q, \Vert v_0 \Vert _{\infty }, T). \, \, \, \, \, \end{aligned}$$

Then:

33 $$\begin{aligned} |v_1^q-v_2^q| \le c_0 |v_1-v_2|, \, \, \, \, |v_1-v_2| \le k_0 |v_1-v_2|_\alpha , \end{aligned}$$

where $k_0(q, \Vert v_0 \Vert _{\infty }, T)$ is a suitable constant.

In the proximity of null critical point any solution converges (this is further shown in the travelling waves analysis to come in the following Section), then it is possible to define $M = \max \lbrace |A+Bv_1|, |A+Bv_2| \rbrace$:

34 $$\begin{aligned} \begin{aligned}{}&\Vert [v_1(A+Bv_1)- v_2(A+Bv_2)]\Vert _\alpha = \int _\mathbb {R} \alpha (\delta ) \sum _{k=0}^4 |D^k[v_1(A+Bv_1)-v_2(A+Bv_2)]|^2 d \delta \\&= \int _\mathbb {R} \alpha (\delta ) \left\{ |v_1(A+Bv_1)-v_2(A+Bv_2)|^2 + \sum _{k=1}^4 |D^k[v_1(A+Bv_1)-v_2(A+Bv_2)]|^2 \right\} d \delta \\&\le M \, 25 \int _\mathbb {R} \alpha (\delta ) \left\{ k_0^2 |(v_1-v_2)|^2 + k_0^2 \sum _{k=1}^4 \sum _{i=1}^k D^k |v_1-v_2|^2 \right\} d \delta \\&= M \, k_0^2 \, 25 \int _\mathbb {R} \alpha (\delta ) \sum _{k=0}^4 |D^k[v_1-v_2]|^2 d \delta = M \, k_0^2 \, 25 \Vert v_1 - v_2\Vert _\alpha . \end{aligned} \end{aligned}$$

As a consequence:

35 $$\begin{aligned} \begin{aligned} \Vert \Sigma _{v_0, \tau }(v_1)-\Sigma _{v_0, \tau }(v_2)\Vert _\alpha&\le M (k_0^2 \, 25 \Vert |y|^\mu \Vert _\alpha + c_0) \int _0^\tau \int _\tau ^s \Vert v_1 - v_2\Vert _\alpha ds d\nu \, \\&= M (k_0^2 \, 25 \Vert |y|^\mu \Vert _\alpha + c_0) \tau (\tau - s) \Vert v_1 - v_2\Vert _\alpha , \end{aligned} \end{aligned}$$

where the term $\Vert |y|^\mu \Vert _\alpha <\infty$ in the norm (Eq. 5). For any ball centered in $0 < \tau \le t$ with radium given by any multiple of $\tau -s$, uniqueness holds in the limit $v_1 \swarrow v_2$ providing a contractive $\Sigma _{v_0, \tau }$, such that $\Sigma _{v_0, \tau }(v_1) \swarrow v_1$ in the space $H_\alpha ^4$. $\square$

## Travelling waves

Travelling waves patterns of solutions are provided by the change $v(y,t) = \Theta (\theta ), \, \theta = y - \lambda t \, \in \mathbb {R}$. Note that $\lambda$ refers to the travelling wave velocity and $\Theta \in L_{loc}^2(\mathbb {R}) \cap L^{\infty }(\mathbb {R})$. As well it may comply with $\Theta \in L^2(\mathbb {R}) \cap L^{\infty }(\mathbb {R}) \cap H_\alpha ^4 (\mathbb {R})$ or $\Theta \in L^2(\mathbb {R}) \cap L^{\infty }(\mathbb {R}) \cap H^ {j=4}(\mathbb {R})$ close the critical point $v = \Theta = 0$.

The Problem (4) is formulated in the travelling wave domain as per the following expression:

36 $$\begin{aligned} - \lambda \Theta ^\prime = - \Theta ^{(4)} + c (\Theta ^q)^\prime + |y|^\mu \Theta (-A-B \, \Theta ). \end{aligned}$$

To control the non-uniform reaction, a bound truncation is defined to the term $|y|^\mu$ as per:

37 $$\begin{aligned} \vert y \vert ^{\mu }_{\epsilon } = \left[ \begin{array}{c} \vert y \vert ^ {\mu } \, \, , \, \, \, 0 \le \vert y \vert < \epsilon \\ \epsilon ^{\mu } \, \, , \, \, \, \vert y \vert \ge \epsilon \end{array} \right] . \end{aligned}$$

After the truncation defined, the following problem is considered (named $P^\prime$):

38 $$\begin{aligned} - \lambda \Theta ^\prime = - \Theta ^{(4)} + c (\Theta ^q)^\prime + |y|^{\mu }_{\epsilon } \Theta (-A-B \Theta ) \le - \Theta ^{(4)} + c (\Theta ^q)^\prime + {\epsilon }^\mu \Theta (-A-B \Theta ). \end{aligned}$$

Note that the truncated term $|y|^{\mu }_{\epsilon }$ is bounded and it permits to define internal balls for $|y| \le \epsilon$. The problem $P^\prime$ can be mentioned as the controlled problem, as the reaction evolves according to the controlling parameter $\epsilon$. Consequently and based on the proposed problem $P^\prime$, the following Lemma, to characterize the travelling wave motion, holds:

### Lemma 3.1

*The travelling wave moves from* $\theta \rightarrow -\infty$ *to* $\theta \rightarrow \infty$ (*i.e*. $\lambda > 0$) *provided*:

39 $$\begin{aligned} c > \frac{\epsilon ^\mu \left( \frac{1}{2} A + \frac{1}{3} B \right) }{q \left( 2 - \frac{1}{q-1} \right) } \end{aligned}$$

where $\epsilon ^\mu > 0$. The travelling wave moves from $\theta \rightarrow \infty$ to $\theta \rightarrow -\infty$ if the speed $\lambda$ is negative, this is $c > \frac{\epsilon ^\mu \left( \frac{1}{2} A + \frac{1}{3} B \right) }{q \left( 2 - \frac{1}{q-1} \right) }$. The travelling wave stops for $c = \frac{\epsilon ^\mu \left( \frac{1}{2} A + \frac{1}{3} B \right) }{q \left( 2 - \frac{1}{q-1} \right) }$.

### **Proof**

First, multiply (Eq. 38) by $\Theta ^\prime$:

40 $$\begin{aligned} -\lambda (\Theta ^\prime )^2 = -\Theta ^{(4)} \Theta ^\prime + c (\Theta ^q)^\prime \Theta ^\prime - {\epsilon }^\mu \, A \Theta \Theta ^\prime - {\epsilon }^\mu \, B \Theta ^2 \Theta ^\prime . \end{aligned}$$

Now, consider the integration along the open domain $(-\infty , \infty )$ in each of the terms. Let us start by the order four derivative:

41 $$\begin{aligned} \begin{aligned} \int \Theta ^{(4)} \Theta ^\prime&= \Theta ^\prime \Theta ^{(3)}- \int \Theta ^{(3)} \Theta ^{(2)} = \Theta ^\prime \Theta ^{(3)} - \left( \Theta ^{(2)} \Theta ^{(2)} - \int \Theta ^{(2)}\Theta ^{(3)}\right) \\&= \Theta ^\prime \Theta ^{(3)} - \Theta ^{(2)} \Theta ^{(2)} + \int \Theta ^{(2)}\Theta ^{(3)} = \Theta ^\prime \Theta ^{(3)} - \Theta ^{{(2)}^{2}} + \Theta ^\prime \Theta ^{(3)} - \int \Theta ^{(4)} \Theta ^\prime . \end{aligned} \end{aligned}$$

As a consequence:

42 $$\begin{aligned} \int \Theta ^{(4)} \Theta ^\prime = \frac{1}{2} \left( 2\Theta ^{\prime \Theta ^{(3)}} - \Theta ^{{(2)}^{2}}\right) . \end{aligned}$$

As pointed, the integral is determined between $-\infty$ and $+\infty$ such that asymptotically, the following holds:

43 $$\begin{aligned} \begin{array}{c} \Theta ^\prime (-\infty ) = \Theta ^{(2)} (-\infty ) = \Theta ^{(3)} (-\infty ) = 0, \\ \\ \Theta ^\prime (\infty ) = \Theta ^{(2)} (\infty ) = \Theta ^{(3)} (\infty ) = 0 . \end{array} \end{aligned}$$

Therefore:

44 $$\begin{aligned} \int _{-\infty }^{\infty } \Theta ^{(4)} \Theta ^\prime = 0. \end{aligned}$$

By standard procedure, it is easy to check that: $\int \Theta \Theta ^\prime = -\frac{1}{2}$.

Consider now the advection term integration:

45 $$\begin{aligned} c \int (\Theta ^q)^\prime \Theta ^\prime = c (\Theta ^q)^\prime \Theta - c \int q(q-1) \Theta ^{q-1} \Theta ^\prime - c q \int \Theta ^q \Theta ^{(2)} . \end{aligned}$$

Note that $\int \Theta ^{q-1} \Theta ^\prime = \Theta ^{q-1} \Theta - \int (\Theta ^{q-1})^\prime \Theta , \, \, \,$ $\int \Theta ^q \Theta ^{(2)} = \Theta ^q \Theta ^{\prime }-\int (\Theta ^q)^\prime \Theta ^{\prime }$. After compilation:

46 $$\begin{aligned} (1-q)c \int (\Theta ^q)^\prime \Theta ^\prime = -cq(q-1)\Theta ^q + cq(q-1) \int \Theta (\Theta ^{q-1})^\prime . \end{aligned}$$

Now, the integral on the left reads:

47 $$\begin{aligned} \int \Theta (\Theta ^{q-1})^\prime = \frac{1}{q-1} \Theta ^q - \Theta ^q, \end{aligned}$$

so that:

48 $$\begin{aligned} c \int _{-\infty }^{\infty } (\Theta ^q)^\prime \Theta ^\prime = cq \left( 2 \Theta ^q - \frac{1}{q-1} \Theta ^q \right) _{-\infty }^{\infty } = c q \left( - 2 + \frac{1}{q-1} \right) . \end{aligned}$$

Eventually, the following integral is assessed:

49 $$\begin{aligned} \int \Theta ^2 \Theta ^\prime = \Theta ^2 \Theta - \int 2 \Theta ^2 \Theta ^\prime \rightarrow \int _{-\infty }^{\infty } \Theta ^2 \Theta ^\prime = \frac{1}{3} \left[ \Theta ^2 \Theta \right] _{-\infty }^{\infty } = \frac{1}{3} (0-1) = - \frac{1}{3}. \end{aligned}$$

Compiling the different integrals assessed:

50 $$\begin{aligned} -\lambda \int (\Theta ^\prime )^2= & {} 0 + c q \left( - 2 + \frac{1}{q-1} \right) + \epsilon ^\mu \left( \frac{1}{2} A + \frac{1}{3} B \right) , \end{aligned}$$

51 $$\begin{aligned} \lambda= & {} \frac{ c q \left( 2 - \frac{1}{q-1} \right) - \epsilon ^\mu \left( \frac{1}{2} A + \frac{1}{3} B \right) }{\int (\Theta ^\prime )^2}. \end{aligned}$$

Note that problem (4) is recovered for $\epsilon \rightarrow \infty$. As $\epsilon > 0$, the Lemma postulations are easily checked by making:

52 $$\begin{aligned} c q \left( 2 - \frac{1}{q-1} \right) - \epsilon ^\mu \left( \frac{1}{2} A + \frac{1}{3} B \right)> 0, \, \, \, \, \rightarrow \, \, \, \, c > \frac{\epsilon ^\mu \left( \frac{1}{2} A + \frac{1}{3} B \right) }{q \left( 2 - \frac{1}{q-1} \right) }. \end{aligned}$$

Idem for < and $=$ in the previous expression. $\square$

### Characterization of travelling waves oscillating behaviour

The pursuit intention consists on showing and characterizing the instability behaviour of travelling waves profiles in the proximity of the null critical state $\Theta = 0$. The proposed approach is inspired by a set of Lemmas and Theorems employed to characterize unstable patterns in Kuramoto–Sivashinsky equation^39^, in a Cahn–Hilliard equation^31^ and in a high and odd order operator^32^. The proposal followed along our analysis varies compared to that in the cited references to account for the problem $P^\prime$ particularities.

#### Lemma 3.2

*Given the Hilbert–Sobolev spaces* $H^4$, $H_\alpha ^4$ *together with the Lebesgue* $L^2$. *The following bounds hold*:

$$\Vert v\Vert _{L^2}^2 \le D_1\Vert v\Vert _{H^4} \, \, , \, \, \, \, \, $$ $$\Vert v\Vert _{L^2}^2 \le D_2\Vert v\Vert _{\alpha }$$.

*This means that any square finite energy profile solution is bounded by the spaces of unstable solutions with derivatives up to fourth order.*

#### **Proof**

Admit the expression (7), then:

53 $$\begin{aligned} \Vert v\Vert _{H^4} = \int _{\mathbb {R}} e^{4 \delta ^2} \, |\hat{v}(\delta , t)|^2 d\delta \ge \inf _{\delta \in (-\infty , \infty )} \lbrace e^{4 \delta ^2} \rbrace \int _{\mathbb {R}} |\hat{v}(\delta , t)|^2 d \, \delta =\Vert v\Vert _{L^2}^2, \end{aligned}$$

as a consequence $D_1 = 1$. The next inequality formulated is shown based on the norm (Eq. 5). Indeed:

54 $$\begin{aligned} \Vert v\Vert _{L^2}^2 \le \int _{\mathbb {R}} \sum _{k=0}^4 |D^k v(\delta )|^2 d \delta \le \int _{\mathbb {R}} \alpha (\delta ) \sum _{k=0}^4 |D^k v(\delta )|^2 d \delta =\Vert v\Vert _\alpha , \end{aligned}$$

a.e. in $\mathbb {R}$. It suffices to consider $D_2 = 1$. $\square$

To show convergence principles in the oscillating travelling wave profile close the null equilibrium $\Theta =0$, the following perturbation function is introduced: $\kappa (y,t) = v(y,t) - \Theta (y,t)$. The problem *P* (Eq. 4) is re-formulated in virtue of the new function $\kappa (y,t)$ and the Travelling wave profile $\Theta (y,t)$ close the condition $\kappa = 0$.

It shall be considered that the involved terms during the perturbation analysis is given only in the derivatives, independently of the order, as any oscillating solution changes the sign of derivatives during evolution. Convergence is, hence, shown in the case of encompassed motion between the actual solution *v* and the travelling wave $\Theta$.

55 $$\begin{aligned} \kappa _t = - \kappa ^{(4)} + \frac{1}{2} \sum _{k = 0}^{\infty } {q \atopwithdelims ()k} (q-k) c \kappa ^{q-k-1} \Theta ^q \kappa _y + \frac{c}{2} \sum _{k = 0}^{\infty } {q \atopwithdelims ()k} \kappa ^{q-k} \Theta ^{q-1} \Theta _y . \end{aligned}$$

Admit the problem:

56 $$\begin{aligned} \kappa _t = L_0\kappa + \Phi (\kappa ), \end{aligned}$$

such that $L_0 = - \partial _y^4$ and $\Phi (\kappa ) = \frac{1}{2} \sum _{k = 0}^{\infty } {q \atopwithdelims ()k} (q-k) c \kappa ^{q-k-1} \Theta ^q \kappa _y + \frac{c}{2} \sum _{k = 0}^{\infty } {q \atopwithdelims ()k} \kappa ^{q-k} \Theta ^{q-1} \Theta _y$.

Note that oscillating profiles are given due to the high order operator. To ensure this occurs, the term $\Phi (\kappa )$ is shown to be bounded in the following Lemma:

#### Lemma 3.3

$\Phi : H^4 \rightarrow L^2$ *is a continuous function. Further, there exist constants* $\varphi _0 > 0$, $\varphi > 1$ *and* $D_3 > 0$ *such that* $\Vert \Phi (\kappa )\Vert _{L^2} \le D_3 \Vert \kappa \Vert _{H^4}^{\varphi } \,$, *provided*: $0<\Vert \kappa \Vert _{H^4}<\varphi _0$.

#### **Proof**

57 $$\begin{aligned} \begin{aligned} \Vert \Phi (\kappa )\Vert _{L^2}&\le \Vert \Phi (\kappa )\Vert _{H^4} \le \frac{c}{2} \sum _{k = 0}^{\infty } {q \atopwithdelims ()k} (q-k) \Vert \kappa ^{q-k-1}\Vert _{H^4} \Vert \Theta ^q\Vert _{\infty } \, \Vert \kappa \Vert _{H^4} \\&+ \frac{c}{2} \sum _{k = 0}^{\infty } {q \atopwithdelims ()k} \Vert \kappa ^{q-k-1}\Vert _{H^4} \Vert \kappa \Vert _{H^4} \Vert \Theta ^{q-1}\Vert _{\infty } \Vert \Theta _y\Vert _{\infty }, \end{aligned} \end{aligned}$$

Then, the following holds:

58 $$\begin{aligned} \Vert \Phi (\kappa )\Vert _{H^4}\le & {} (\Vert \Theta ^q\Vert _{\infty } + \Vert \Theta ^{q-1}\Vert _{\infty } \Vert \Theta _y\Vert _{\infty }) \frac{c}{2} \sum _{k = 0}^{\infty } {q \atopwithdelims ()k} (q-k) \Vert \kappa \Vert _{H^4}^{-k-1} \Vert \kappa \Vert _{H^4}^{q+1} . \end{aligned}$$

59 $$\begin{aligned} \Vert \Phi (\kappa )\Vert _{L^2}\le & {} \Vert \Phi (\kappa )\Vert _{H^4} \le D_3 \Vert \kappa \Vert _{H^4}^{\varphi }, \end{aligned}$$

such that $\varphi = q+1$ and $D_3 = (\Vert \Theta ^q\Vert _{\infty } + \Vert \Theta ^{q-1}\Vert _{\infty } \Vert \Theta _y\Vert _{\infty }) \frac{c}{2} \sum _{k = 0}^{\infty } {q \atopwithdelims ()k} (q-k) M$, for a sufficiently large $M \ge \sum _{k = 0}^{\infty } \Vert \kappa \Vert _{H^4}^{-k-1}$. $\square$

It shall be noted that the previous Lemma can be proved following a similar procesure for $\Phi : H_{\alpha } \rightarrow L^2$, provided there exist $\varphi > 1$, $r > 0$ and $D_4 > 0$ such that $\Vert \Phi (\kappa )\Vert _{L^2} < D_4 \Vert \kappa \Vert _\alpha ^{\varphi } \,$, for $0<\Vert \kappa \Vert _{\varphi }<r$. In this last case $D_4 = (\Vert \Theta ^q\Vert _{\infty } \theta + \Vert \Theta ^{q-1}\Vert _{\infty } \Vert \Theta _y\Vert _{\infty }) \frac{c}{2} \sum _{k = 0}^{\infty } {q \atopwithdelims ()k} (q-k) M_1$, for a large $M_1 \ge \sum _{k = 0}^{\infty } \Vert \kappa \Vert _{\alpha }^{-k-1}$.

Note that $\Sigma _{\kappa _0, \tau }$ has been proved as a bounded operator in Theorem 2.1. Furthermore, the mapping operator $\Sigma _{\kappa _0, \tau }$ is the infinitesimal generator of a strongly continuous semi-group that admits an exponential representation to the linear operator $L_0$. Based on this, the following Lemma enunciates:

#### Lemma 3.4

*The heterogeneous diffusion given by linear* $L_0$ *generates a strongly continuous semigroup abstractly represented by* $e^{tL_0}$ *that satisfies*:

60 $$\begin{aligned} \int _0^1 \Vert e^{tL_0}\Vert _{L^2 \rightarrow H^4} \, dt = E_1< \infty , \, \, \, \, \, \, \, \, \, \, \, \, \, \int _0^1 \Vert e^{tL_0}\Vert _{L^2 \rightarrow H_\alpha ^4} \, dt = E_2 < \infty \end{aligned}$$

#### **Proof**

First, and given the fundamental problem $\kappa _t = - \kappa ^{(4)}$, the following exponential solution holds in the Fourier domain (*f*):

61 $$\begin{aligned} \hat{\kappa }(f, t) = e^{-tf^4} \hat{\kappa }_0(f). \end{aligned}$$

Then:

62 $$\begin{aligned} \Vert \kappa \Vert _{H^4}\le & {} \Vert e^{-tf^4}\Vert _{H^4} \, \Vert \kappa _0\Vert _{H^4} \le \sup _{f \in (-\infty , \infty )} \lbrace e^{4 f^2 - 2 t f^4} \rbrace \Vert \kappa _0\Vert _{L^2}^2, \end{aligned}$$

63 $$\begin{aligned} \Vert e^{-tf^4}\Vert _{L^2 \rightarrow H^4} \Vert \kappa _0\Vert _{L^2}^2\le & {} \Vert e^{-tf^4}\Vert _{H^4} \Vert \kappa _0\Vert _{H^4} \le \sup _{f \in (-\infty , \infty )} \lbrace e^{4 f^2 - 2 t f^4} \rbrace \Vert \kappa _0\Vert _{L^2}^2. \end{aligned}$$

Then:

64 $$\begin{aligned} \Vert e^{-tf^4}\Vert _{L^2 \rightarrow H^4} \le \sup _{f \in (-\infty , \infty )} \lbrace e^{4 f^2 - 2 t f^4} \rbrace = e^{2 t^{-1}} \, , \, \, \, \, \, \, 0<t\le 1. \end{aligned}$$

A finite $E_1$ holds upon integration with *t* in (0, 1]. Proceeding in a similar way for $E_2$:

65 $$\begin{aligned} \begin{aligned} \Vert \kappa \Vert _\alpha&= \int _{\mathbb {R}} \alpha (\delta ) \sum _{k=0}^4 |D^k \hat{\kappa }(\delta )|^2 d \, \delta \le 2 \Upsilon \int _{\mathbb {R^+}} e^{a_0 \delta ^{4/3}} |\hat{\kappa }(\delta )|^2 d\delta \\&\le 2 \Upsilon \int _{\mathbb {R^+}} e^{a_0 \delta ^{4/3}- 2 t \delta ^4} |\hat{\kappa }_0(\delta )|^2 d\delta \le 2 \Upsilon \sup _{\delta \in (0, \infty )} \lbrace e^{a_0 \delta ^{4/3}-2 t \delta ^4} \rbrace \Vert \kappa _0\Vert _{L^2}^2 = e^{B_c \, a_0^{3/2} t^{-1/2}} \Vert \kappa _0\Vert _{L^2}^2, \end{aligned} \end{aligned}$$

being $B_c > 0$ a suitable constant. Note $\Vert e^{-t\delta ^4}\Vert _{L^2 \rightarrow H_\alpha ^4} \Vert \kappa _0\Vert _{L^2}^2 \le \Vert \kappa \Vert _\alpha$. In addition, the following holds:

66 $$\begin{aligned} \Vert e^{-t\delta ^4}\Vert _{L^2 \rightarrow H_\alpha ^4} \le e^{B_c \, a_0^{3/2} t^{-1/2}}. \end{aligned}$$

A finite $E_2$ holds after integration with regards to *t* in (0, 1]. $\square$

The next intention is to characterize the spectrum of *L* (as defined in expression (9) and for $N=1$) close the null critical point. To this end, the following Lemma enunciates:

#### Lemma 3.5

*The local (local means close the equilibrium null state) spectrum of* *L* *(see expressions* (8) *and* (9)) *in* $H^4$ *has at least one eigenvalue* (*s*) *such that* $Re(s)>0$.

#### **Proof**

The spectrum of *L* in the proximity of the null critical state may be shown making use of Evans functions (see^40^ for a detail review of such theory and the relation with the characteristic polynomial of a matrix). In the presented analysis, the Eq. (38) is firstly converted into a matrix representation and an homotopy graph is provided close the null critical point to study the influence of the travelling wave speed.

67 $$\begin{aligned} \left( \begin{array}{l} \Theta _1 \\ \Theta _2 \\ \Theta _3 \\ \Theta _4 \end{array}\right) ^\prime = \left( \begin{array} {llll} 0 &{} 1 &{} 0 &{} 0 \\ 0 &{} 0 &{} 1 &{} 0 \\ 0 &{} 0 &{} 0 &{} 1 \\ (-A-B \, \Theta _1)|y|^\mu _\epsilon &{} \lambda +cq\Theta _1^{q-1} &{} 0 &{} 0\\ \end{array} \right) \left( \begin{array}{l} \Theta _1 \\ \Theta _2 \\ \Theta _3 \\ \Theta _4 \end{array}\right) \end{aligned}$$

68 $$\begin{aligned} \Theta ^\prime _M= \Xi (c,a,\lambda , x) \Theta _M. \end{aligned}$$

The characteristic polynomial for the matrix $\Xi$ is:

69 $$\begin{aligned} R(s) = s^4 - (cq\Theta _1^{q-1}+\lambda )s + (A + B \Theta _1)|y|^\mu _\epsilon = 0. \end{aligned}$$

**Figure 1:**
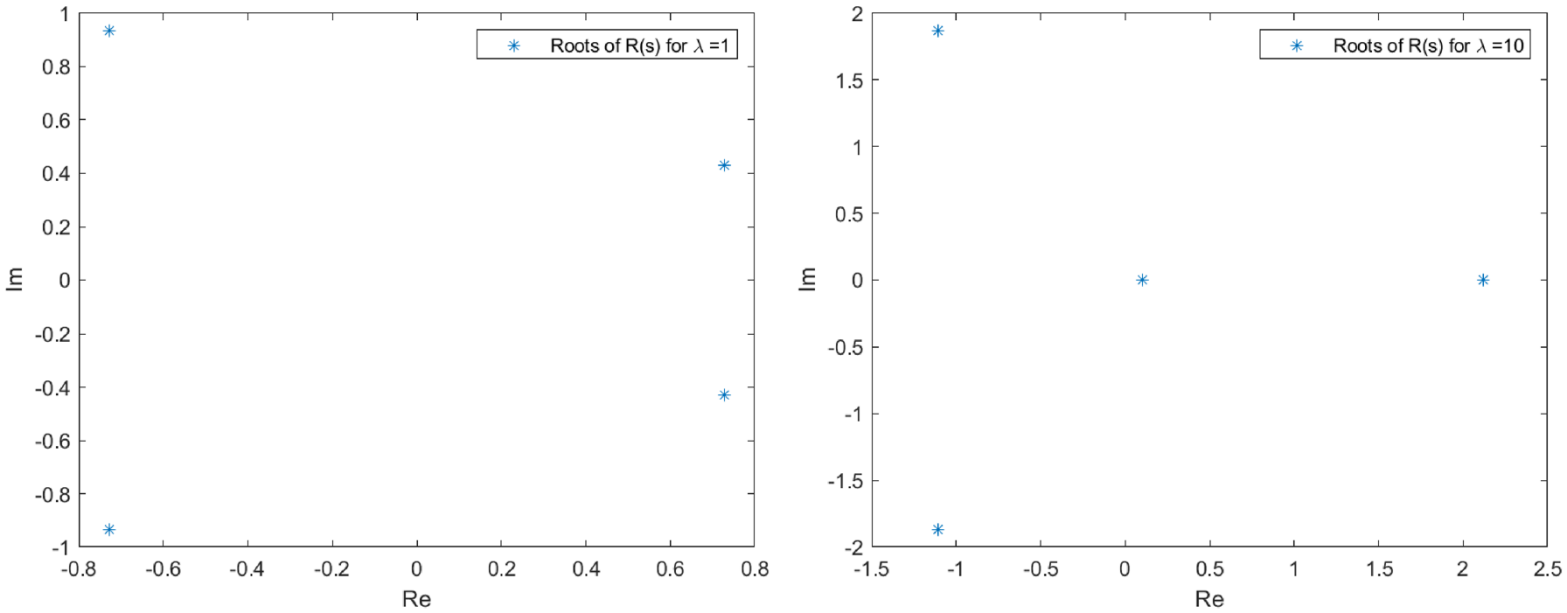
Complex domain (real part—horizontal axis, imaginary part—vertical axis) for $\lambda = 1$ (left) and $\lambda = 10$ (right). Evolution of *R*(*s*) roots in the complex domain considering $\Theta _1 < \epsilon \rightarrow 0$ and $|y|^\mu _\epsilon =1$. Note that at least one root has $Re(s)>0$.

**Figure 2:**
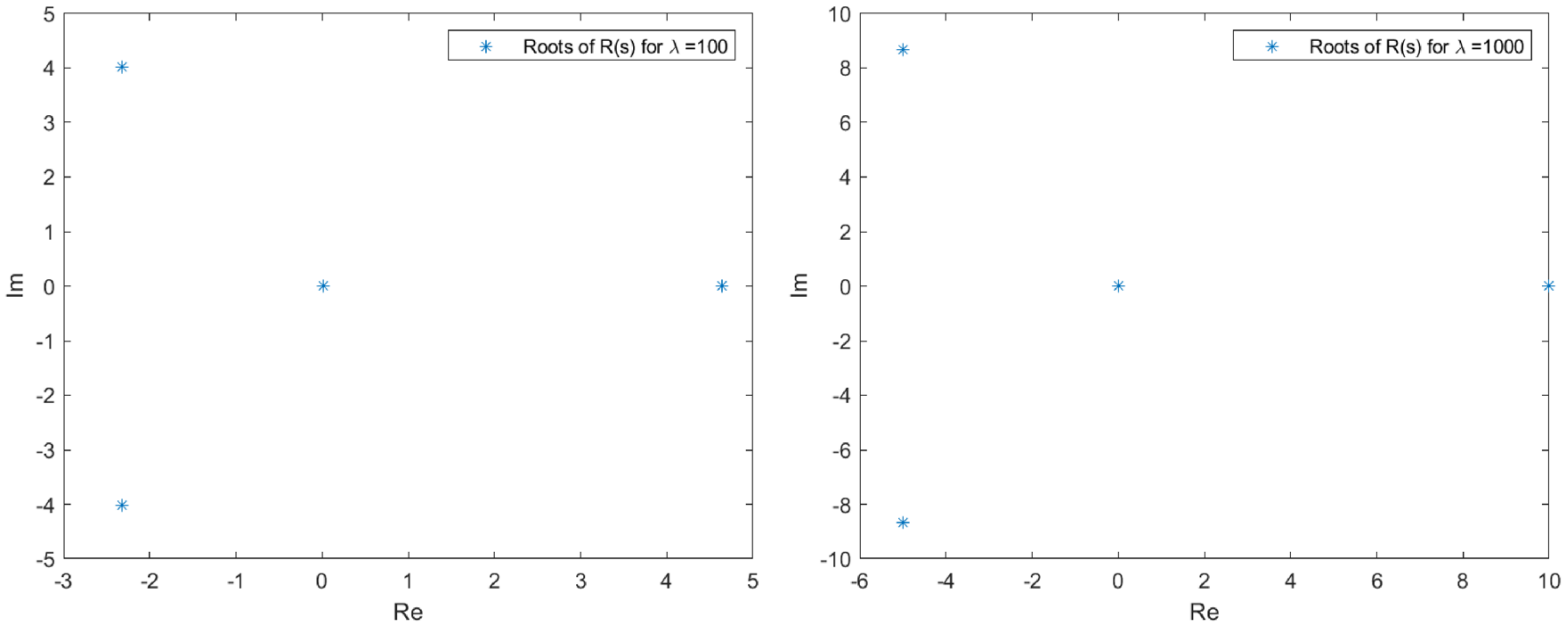
Complex domain (real part—horizontal axis, imaginary part—vertical axis) for $\lambda = 100$ (left) and $\lambda = 1000$ (right). Evolution of *R*(*s*) roots in the complex domain considering $\Theta _1 < \epsilon \rightarrow 0$ and $|y|^\mu _\epsilon =1$. Note that at least one root has $Re(s)>0$.

**Figure 3:**
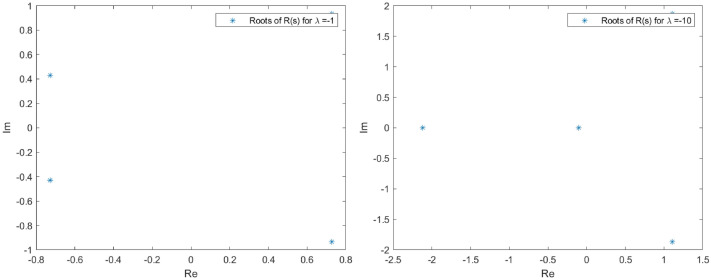
Complex domain (real part—horizontal axis, imaginary part—vertical axis) for $\lambda = -1$ (left) and $\lambda = -10$ (right). Evolution of *R*(*s*) roots in the complex domain considering $\Theta _1 < \epsilon \rightarrow 0$ and $|y|^\mu _\epsilon =1$. Note that at least one root has $Re(s)>0$.

**Figure 4:**
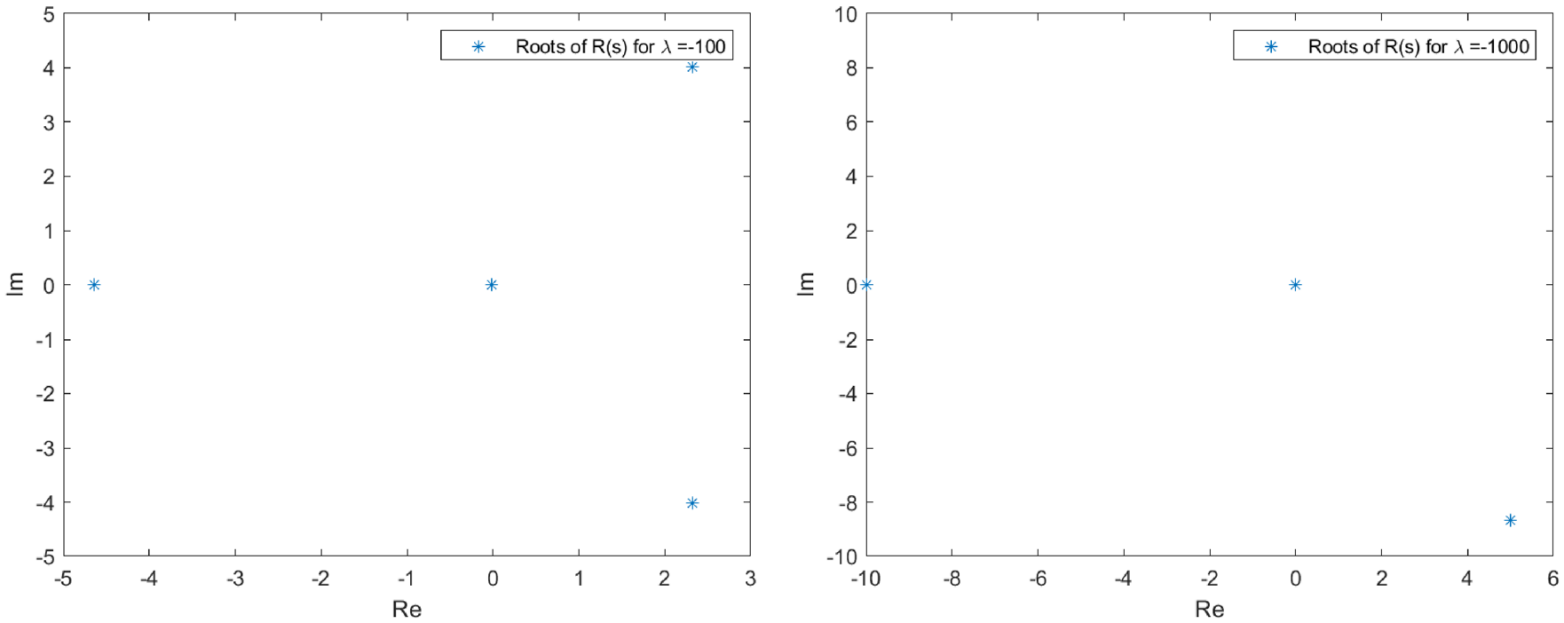
Complex domain (real part—horizontal axis, imaginary part—vertical axis) for $\lambda = -100$ (left) and $\lambda = -1000$ (right). Evolution of *R*(*s*) roots in the complex domain considering $\Theta _1 < \epsilon \rightarrow 0$ and $|y|^\mu _\epsilon =1$. Note that at least one root has $Re(s)>0$.

Admit the approach $\Theta _1 < \epsilon \rightarrow 0$, then it is easy to check that *R*(*s*) has at least one root such that $Re(s)>0$. Indeed, given an arbitrary $\epsilon$ in the expression $|y|^\mu _\epsilon$, for $0<s<<1$, $R(s)<0$ while for $s>> 1$, $R(s)>0$. The roots behaviour to *R*(*s*) can be guessed admitting $|y|^\mu _\epsilon =1$ with no loss of generality (refer to Figs. 1, 2, 3 and 4 for the root evolution and different values in the travelling wave speeds. Such figures represent the homotopy graphs to determine the influence of the speeds). $\square$

### Geometric perturbation theory

Firstly, consider the following fundamental manifold defined in accordance with expression (38):

70 $$\begin{aligned} M_0 = \lbrace \phi _1 , \phi _2, \phi _3, \phi _4 \, / \, \, \phi _1^\prime = \phi _2 \, , \, \phi _2^\prime = \phi _3 \, , \, \phi _3^\prime = \phi _4 \, , \, \phi _4^\prime = \lambda \phi _2 + c q \phi _1^{q-1}\phi _2 + |\theta + \lambda t|^\mu \phi _1 (-A-B\phi _1) \rbrace \, . \end{aligned}$$

Such the equilibrium is given at the point (0, 0, 0, 0). Admit the following perturbed manifold, $M_{\epsilon }$, in the vicinity of the critical point (0, 0, 0, 0):

71 $$\begin{aligned} M_{\epsilon } = \lbrace \phi _1 \sim |\epsilon |<<1 , \phi _2, \phi _3, \phi _4 \, / \, \, \phi _1^\prime = \phi _2 \, , \, \phi _2^\prime = \phi _3 \, , \, \phi _3^\prime = \phi _4 \, , \, \phi _4^\prime = \lambda \phi _2 + c q \epsilon ^{q-1}\phi _2 \rbrace \, . \end{aligned}$$

The manifold $M_\epsilon$ shall be shown as a normal hyperbolic. To this end, the Fenichel invariant manifold Theorem^41^ is considered as established in^42,43^. The eigenvalues of $M_\epsilon$ in the proximity of the critical point, and in the transversal direction to the tangent space shall have non-zero real part. After computation, the eigenvalues for $M_\epsilon$ are given as:

72 $$\begin{aligned} \left( 0, \left( \lambda + cq\epsilon ^{q-1} \right) ^{\frac{1}{3}}, \left( \lambda + cq\epsilon ^{q-1} \right) ^{\frac{1}{3}} e^{i 2\varrho /3}, \left( \lambda + cq\epsilon ^{q-1} \right) ^{\frac{1}{3}} e^{i 4\varrho /3} \right) . \end{aligned}$$

The eigenvector to the zero eigenvalue is $[\epsilon ,0, 0, 0]$ that is tangent to $M_\epsilon$. Therefore, all the eigenvalues transversal to $M_\epsilon$ have non-zero real part leading to state that $M_\epsilon$ is a hyperbolic manifold.

Now, the manifold $M_{\epsilon }$ shall be proved to be locally invariant under the flows defined (particularly considering the flow for $\phi _4^\prime$). To this end and according to reference^43^ it shall be shown that for all $r>0$, for all open interval *J* with a constant $a \in J$ and for any given value of $i \in \mathbb {N}$, there exists a $\delta$ such that for $\epsilon \in (0, \delta )$, the manifold $M_{\epsilon }$ is kept invariant. To this end, admit $i \ge 1$ and the following set of functions:

73 $$\begin{aligned} \begin{array}{c} \phi ^{M_0} =\lambda \phi _2 + c q \phi _1^{q-1}\phi _2 + |\theta + \lambda t|^\mu \phi _1 (-A- B \, \phi _1), \\ \phi ^{M_\epsilon } = \lambda \phi _2 + c q \epsilon ^{q-1}\phi _2. \end{array} \end{aligned}$$

which are $C^i (\overline{B_r (0)} \times \overline{J} \times [0, \delta ])$ close the null critical state.

A value for $r>0$ can be selected in the consideration that $M_\epsilon \cap B_r(0)$ is a sufficiently large interval so as to permit the evolution of the travelling wave profile along its domain. The assessment of $\delta$ is based on determining the distance between the flows for $M_{\epsilon }$ and $M_0$. To this end, consider that the involved functions in each flow are measurable (at least a.e.) in $B_r(0)$ with the norm (Eq. 5):

74 $$\begin{aligned} \Vert \phi ^{M_0}-\phi ^{M_{\epsilon }}\Vert _{\alpha } \le cq \Vert \phi _1^{q-1}-\epsilon ^{q-1}\Vert _{\alpha } \Vert \phi _2\Vert _{\alpha } + |\theta + \lambda t|^\mu \Vert \phi _1\Vert _{\alpha } \Vert A + B \phi _1\Vert _{\alpha }, \end{aligned}$$

In the null critical state, it can be considered the asymptotic approach $\phi _2=0$ such that for a small $\phi _1$:

75 $$\begin{aligned} \Vert \phi ^{M_0}-\phi ^{M_{\epsilon }}\Vert _{\alpha } \le \left( cq \Vert \phi _1^{q-1}-\epsilon ^{q-1}\Vert _{\alpha } + |\theta + \lambda t|^\mu \Vert A + B \phi _1\Vert _{\alpha } \right) \Vert \phi _1\Vert _{\alpha }. \end{aligned}$$

For $\phi _1< |\epsilon |<< 1$, admit the definition of $\delta (t) = cq \Vert \phi _1^{q-1}-\epsilon ^{q-1}\Vert _{\alpha } + |\theta + \lambda t|^\mu \Vert A + B \phi _1\Vert _{\alpha }$, for each *t* in $B_r(0)$. Under these conditions, $\delta$ is finite and consequently $M_\epsilon$ is close $M_0$ to preserve the normal hyperbolic condition.

**Figure 5:**
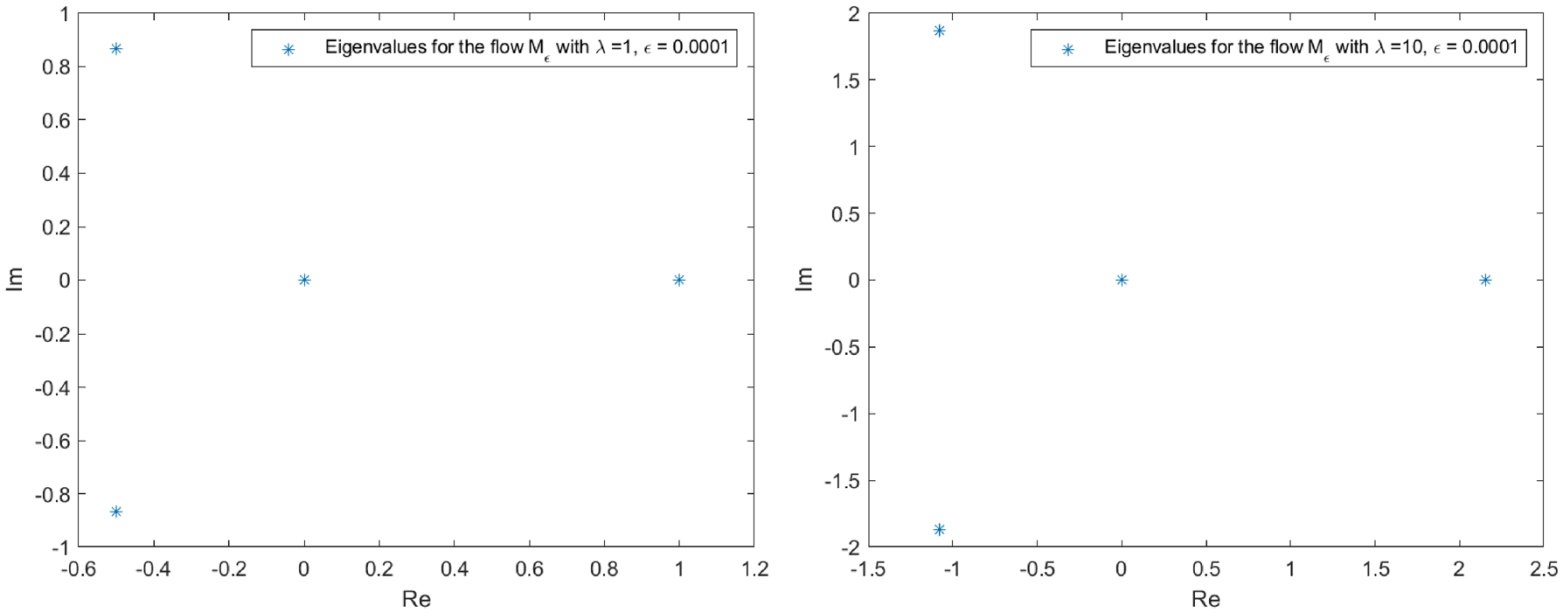
$M_\epsilon$ eigvenalues evolution for $\lambda = 1$ (left) and $\lambda = 10$ (right). Note that $c=1$ and $q=2$. A solution in $B_r(0)$ can be obtained by linear combination of the exponential bundles associated to each eigenvalue.

**Figure 6:**
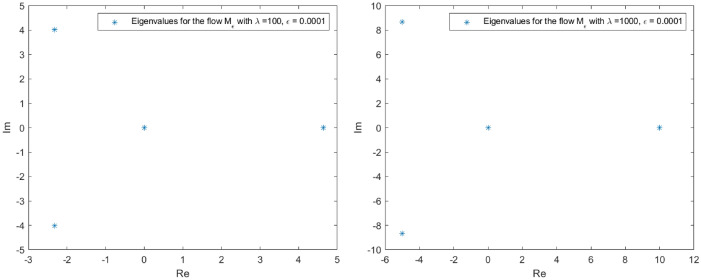
$M_\epsilon$ eigvenalues evolution for $\lambda = 100$(left) and $\lambda = 1000$ (right). Note that $c=1$ and $q=2$. A solution in $B_r(0)$ can be obtained by linear combination of the exponential bundles associated to each eigenvalue.

Note that the linearized flows in the invariant manifold $M_\epsilon$ permit to determine asymptotic travelling wave profiles in $B_r(0)$ by direct computation of a linear system of ODEs and given specific values in the involved parameters *c*, *q* and $\lambda$ (see Figs. 5 and 6).

### Construction of travelling wave profiles

The existence and construction of travelling wave profiles can be made considering the common transversality between the manifolds $M_\epsilon$ and $M_\mu$. For each manifold, a profile is obtained in the asymptotic condition and close the null critical point.

As represented in Figs. 5 and 6 , it is possible to conclude on the existence of complex conjugate eigenvalues leading to oscillating bundles of solution. This suggests that the oscillating property is inherent to our problem and exists for any searched solutions. One of the main consequences of this last statement is the impossibility to find an appropriate travelling wave speed for which oscillations vanish in accordance with the Fisher-KPP philosophy introduced in^17^. Alternatively, the exploration of a specific speed for which the profile is positive can be translated into searching an appropriate speed for which the first travelling wave minimum is positive. Probably, this may happen only locally in time given the non-uniform reaction term.

The obtaining process of travelling waves profiles is performed with the solver bvp4c in the Sofware MATLAB. This solver is based on an implicit Runge–Kutta with interpolant extensions. In addition, the solver employs a collocation method to build the travelling profiles for particular conditions at the borders^36^. In our analysis, we consider a step like function as initial data $\Theta _0 (\theta ) = \, H(-\theta )$. This function enables us to study the evolution of a positive mass (at $\theta < 0$) and a null one (for $\theta > 0$). Furthermore, a heteroclinic orbit holds between the two states as $\Theta _0 (\theta <0) = 1$ and $\Theta _0 (\theta >0) = 0$. To vanish the influence of the pseudo-boundary, required by the collocation procedure, the integration domain is admitted as large, i.e. $\theta \in (-1000, 1000)$.

Our main target is to explore the existence of a value in the scaled variable $\varrho = \lambda t$ for which the first travelling minimum is positive. For the sake of simplicity and without loss of generality, the numerical scheme has been obtained for $\lambda = 1$, introducing a wide interval of *t* values until the first minimum in the profile is positive. The numerical exploration suggests that in virtue of the non-uniform reaction, it is not possible to find a unique travelling wave speed (valid for any time) for which the first minimum is positive, and hence, ensuring a stationary inner region of positive solutions. Consequently, any positive region is explored only locally in time.

**Figure 7:**
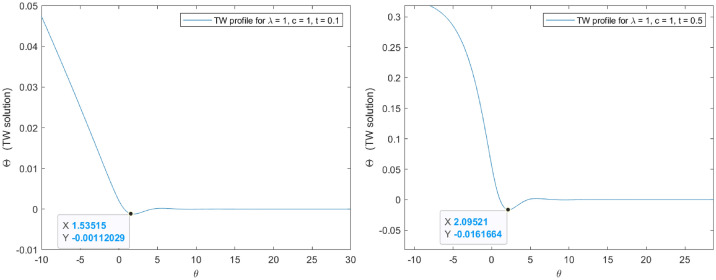
Exact travelling wave profiles for $A=B=1$. Note that the first minimum is negative and the profiles evolves oscillating.

**Figure 8:**
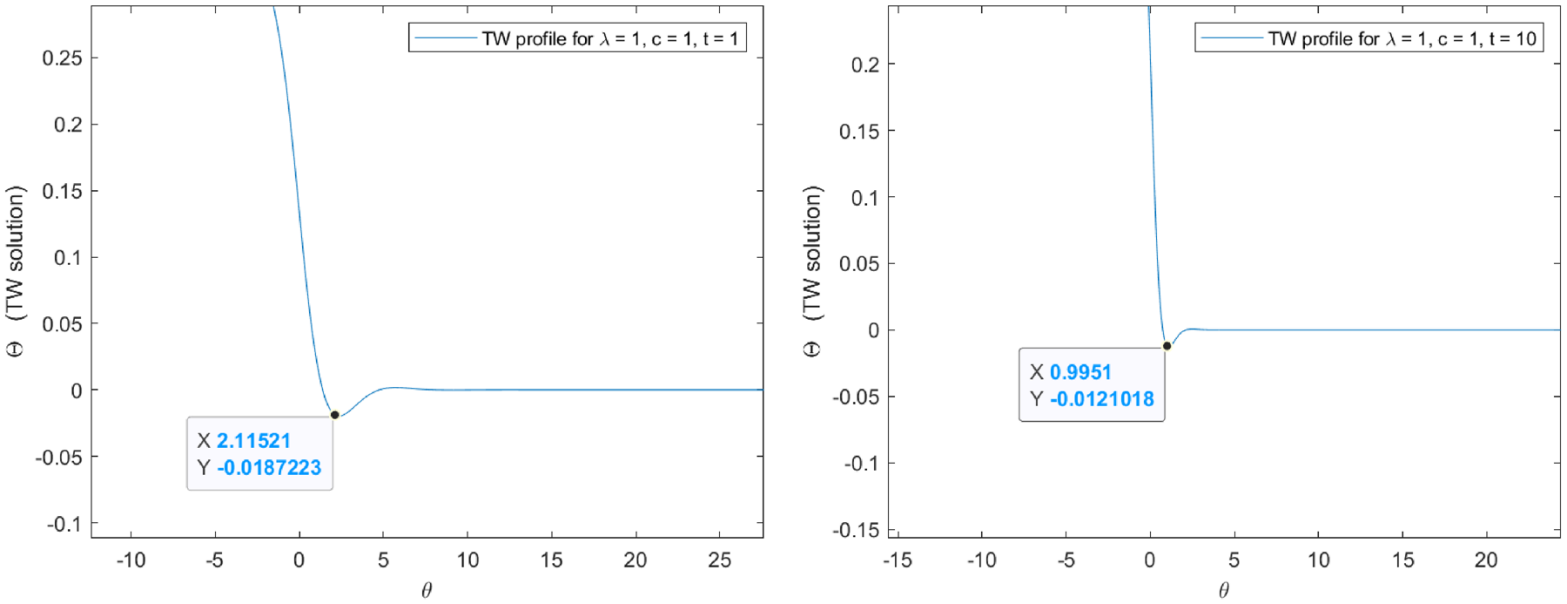
Exact travelling wave profiles for $A=B=1$. Note that the first minimum is negative and the profiles evolves oscillating.

The numerical assessments have been pursuit for specific values in the involved parameters (see Figs. 7 and 8 ). In particular, $c = 1$, $q = 2$, $A = B = 1$ and $\mu = 2$. Note that for the selected values the convection coefficient complies with expression (39), then the profile moves from the left to the right.

**Figure 9:**
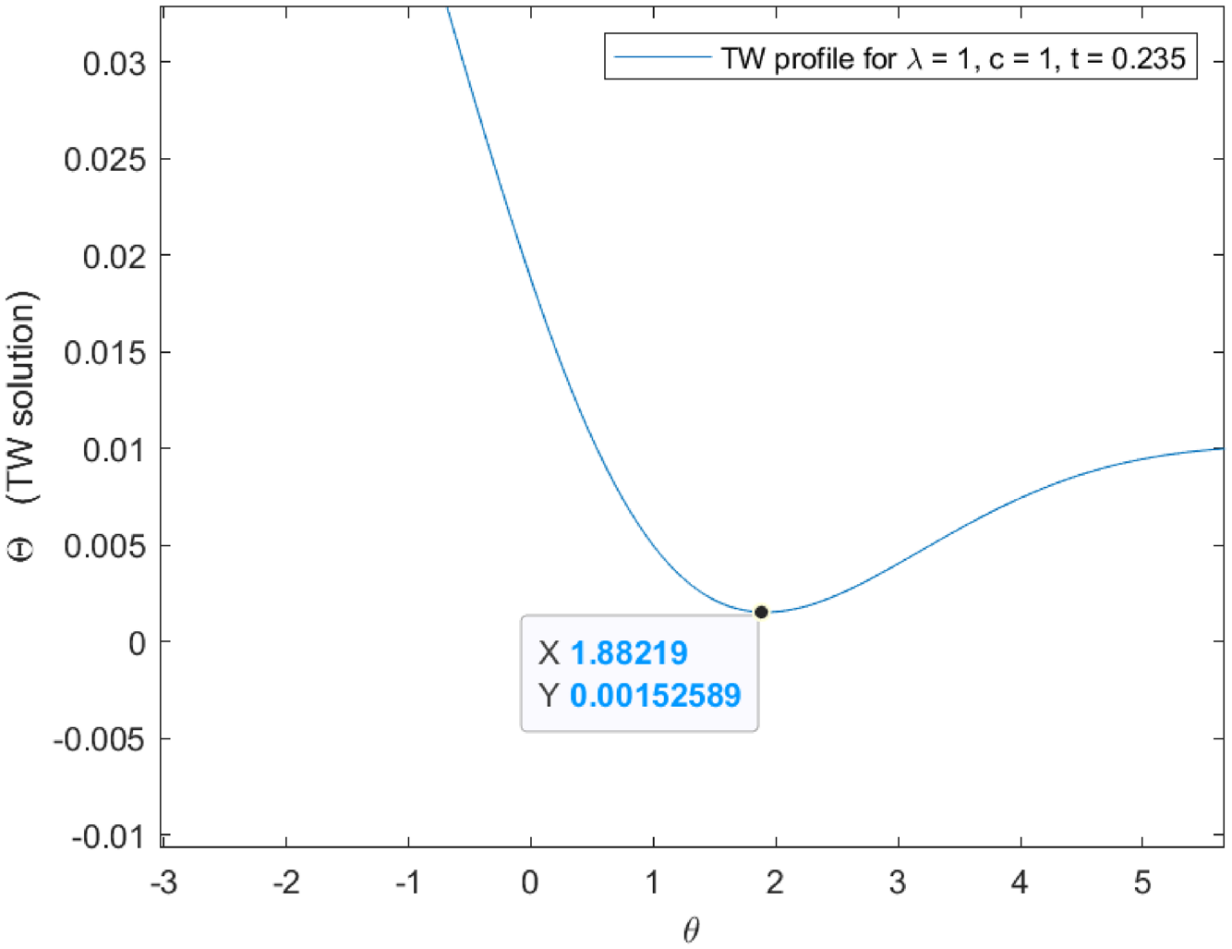
The first minimum is positive in $t = 0.235$. For lower or higher $t-$values the first minimum is negative.

Considering the first minimum positivity in Fig. 9, it is possible to obtain a spatial region of purely monotone solutions and for which a maximum principle holds locally in time. This spatial inner region is assessed based on the localization of $\theta = 1.88219$, the local time $t = 0.235$ and the travelling wave change $\theta = y - \lambda t$, such that:

76 $$\begin{aligned} |y| = 2.117 \end{aligned}$$

## Conclusions

The proposed analysis has been focused on the mathematical treatment of an Extended Darcy–Forchheimer flow. Such extended problem has been introduced based on mathematical principles, having been such mathematical scope the main target of the presented study. To this end, we started by the definition of generalized functional spaces to characterize the oscillating behaviour of the high order operator. To this end, the $H_\alpha ^4$ Hilbert–Sobolev space has permitted to account for the properties of solutions up to its fourth order derivative. In addition, the mollifier space $H^j$ has permitted to account for global mollification properties in the exponential mollifying-kernel. Afterwards, the problem has been formulated making use of an abstract evolution, such that existence and uniqueness of solutions are shown upon and based on the generalized Hilbert–Sobolev spaces defined. Solutions have been shown to be inherently unstable in virtue of the high order operator. To this end, a certain series of Lemmas (from Lemma 3.2 to 3.5) have been shown to apply in our case. Finally, a numerical exploration has been promoted in the search of travelling waves profiles and regions where positivity holds. In this sense and as a main finding, it has been observed that solutions are oscillating in the proximity of the null critical point (and hence being negative since the first minimum) except for a local time that has been precised assessed for specific values in the problem parameters ($c = 1$, $q = 2$, $A = B = 1$ and $\mu = 2$). It shall be noted that the numerical explorations have been provided for certain values in the involved parameters *p*, *c*, *q*, *A* and *B* but conclusions remain valid for any other possible combination. i.e. a spatial region where solutions are positive since the first minimum only holds locally in time.

## Data Availability

The author states that all data used is accessible.

## References

[1] Hayat T, Iqbal Z, Qasim M, Obaidat S (2012). Steady flow of an Eyring Powell fluid over a moving surface with convective boundary conditions. Int. J. Heat Mass Transf..

[2] Javed T, Abbas Z, Ali N, Sajid M (2013). Flow of an Eyring-Powell nonnewtonian fluid over a stretching sheet. Chem. Eng. Commun..

[3] Khan JA (2014). On model for three-dimensional flow of nanofluid: An application to solar energy. J. Mol. Liq..

[4] Rasool G (2020). Entropy generation and consequences of binary chemical reaction on MHD Darcy-Forchheimer Williamson nanofluid flow over non-linearly stretching surface. Entropy.

[5] Ara A, Khan NA, Khan H, Sultan FF (2014). Radiation effect on boundary layer flow of an Eyring-Powell fluid over an exponentially shrinking sheet. Ain-Shams Eng. J..

[6] Dero S (2021). Influence of a Darcy-Forchheimer porous medium on the flow of a radiative magnetized rotating hybrid nanofluid over a shrinking surface. Sci. Rep..

[7] Al-Kouz W (2021). MHD Darcy-Forchheimer nanofluid flow and entropy optimization in an odd-shaped enclosure filled with a (MWCNT-Fe3O4/water) using galerkin finite element analysis. Sci. Rep..

[8] Hayat T, Haider F, Muhammad T, Alsaedi A (2017). On Darcy-Forchheimer flow of carbon nanotubes due to a rotating disk. Int. J. Heat Mass Transf..

[9] Hayat T, Rafique K, Muhammad T, Alsaedi A, Ayub M (2018). Carbon nanotubes significance in Darcy-Forchheimer flow. Results Phys..

[10] Jawad M, Shah Z, Islam S, Bonyah E, Khan ZZ (2018). Darcy-Forchheimer flow of MHD nanofluid thin film flow with Joule dissipation and Navier partial slip. J. Phys. Commun..

[11] Kieu, T. Existence of a solution for generalized Forchheimer flow in porous media with minimal regularity conditions. *J. Math. Phys.* **61**(1), (2020).

[12] Rasool G (2020). Entropy generation and consequences of MHD in DarcyForchheimer nanofluid flow bounded by non-linearly stretching surface. Symmetry.

[13] M.A. & Hayat, T. DarcyForchheimer flow of magneto Maxwell liquid bounded by convectively heated sheet. *Results Phys.* **6**, 884–890 (2016).

[14] Saif RS, Hayat T, Ellahi T, Muhammad T, Alsaedi A (2019). Darcy-Forchheimer flow of nanofluid due to a curved stretching surface.

[15] Saif RS, Muhammad T, Sadia H (2020). Significance of inclined magnetic field in Darcy-Forchheimer flow with variable porosity and thermal conductivity. Physica A Stat. Mech. Appl..

[16] Sajid T, Sagheer M, Hussain S, Bilal M (2018). Darcy-Forchheimer flow of Maxwell nanofluid flow with nonlinear thermal radiation and activation energy. AIP Adv..

[17] Kolmogorov AN, Petrovskii IG, Piskunov NS (1937). Study of the Diffusion Equation with Growth of the Quantity of Matter and Its Application to a Biological Problem 1.

[18] Fisher RA (1937). The advance of advantageous genes. Ann. Eugenics.

[19] Aronson, D. Density-dependent interaction-diffusion systems. in *Proceedings of the Advanced Seminar on Dynamics and Modeling of Reactive System*. (Academic Press, 1980).

[20] Aronson D, Weinberger H (1975). Nonlinear Diffusion in Population Genetics, Combustion and Nerve Propagation 5–49.

[21] Aronson D, Weinberger H (1978). Multidimensional nonlinear diffusion arising in population genetics. Adv. Math..

[22] Ladyzhenskaya, O. Some results on modifications of three-dimensional Navier-Stokes equations. in*Nonlinear Analysis and Continuum Mechanics*. 73–84 (1998).

[23] Cohen DS, Murray JD (1981). A generalized diffusion model for growth and dispersal in a population. J. Math. Biol..

[24] Dee, G.T. & Van Sarloos, W. Bistable systems with propagating fronts leading to pattern formation. *Phys. Rev. Lett.* **60** (1998). 10.1103/PhysRevLett.60.2641 10038411

[25] Peletier, L.A. & Troy, W.C. Spatial patterns. Higher order models in physics and mechanics. in *Progress in Non Linear Differential Equations and Their Applications*. Vol. 45. (Université Pierre et Marie Curie, 2001).

[26] Bonheure D, Sánchez L (2006). Heteroclinics Orbits for some classes of second and fourth order differential equations. Handb. Differ. Equ..

[27] Bonheure D, Hamel F (2017). One-dimensional symmetry and Liouville type results for the fourth order Allen-Cahn equation in $$\mathbb{R}$$. Chin. Ann. Math. Ser. B.

[28] Audrito, A. & Vázquez, J.L. The Fisher-KPP problem with doubly nonlinear “fast” diffusion *Nonlinear Anal.* **157**, 212–248 (2017).

[29] Díaz JL (2022). Non-Lipschitz heterogeneous reaction with a p-Laplacian operator. AIMS Math..

[30] Montaru A (2014). Wellposedness and regularity for a degenerate parabolic equation arising in a model of chemotaxis with nonlinear sensitivity. Discrete Continuous Dyn. Syst. B.

[31] Gao Hongjun, Liu Changchun (2004). Instabilities of traveling waves of the convective-diffusive Cahn-Hilliard equation. Chaos Solit. Fractals.

[32] Li Z, Liu C (2012). On the nonlinear instability of traveling waves for a sixth-order parabolic equation. Abstr. Appl. Anal..

[33] Galaktionov VA (2001). On a spectrum of blow-up patterns for a higher-order semilinear parabolic equation.

[34] Nadin, G. & Rossi L. Transition waves for Fisher-KPP equations with general time-heterogeneous and space-periodic coefficients. https://arxiv.org/pdf/1603.00428.pdf (2016).

[35] Galaktionov, V. *Towards the KPP-Problem and Log-Front Shift for Higher-Order Nonlinear PDEs I* (Cornwell University, Bi-Harmonic and Other Parabolic Equations) arXiv:1210.3513 (2012).

[36] Enright, H. & Muir P.H. *A Runge-Kutta type boundary value ODE soLwer with defect control.* in *Technical Report 267/93, University of Toronto, Department of Computer Sciences*. (1993).

[37] Goldshtein, V. Ukhlov. & A,. Weighted Sobolev Spaces and embeddings Theorems. *Trans. Am. Math. Soc.* **361**, 3829–3850 (2009).

[38] Kesavan, S. *Topics in Functional Analysis and Applications*. (New Age International (formerly Wiley-Eastern), 1989).

[39] Strauss W, Wang G (2002). Instabilities of travelling waves of the Kuramoto-Sivashinsky equation. Chin. Ann. Math. B..

[40] Alexander J, Gardner R, Jones C (1990). A topological invariant arising in the stability analysis of travelling waves. J. Reine Angew. Math..

[41] Fenichel N (1971). Persistence and smoothness of invariant manifolds for flows. Indiana Univ. Math. J..

[42] Akveld ME, Hulshof J (1998). Travelling wave solutions of a fourth-order semilinear diffusion equation. Appl. Math. Lett..

[43] Jones, C.K.R.T. & C.K. *Geometric Singular Perturbation Theory in Dynamical Systems* (Springer, 1995).

